# Building and Utilizing a Digital Platform to Strengthen Preparedness of Undergraduates Volunteering With the Hospital Elder Life Program

**DOI:** 10.1111/jgs.70436

**Published:** 2026-04-15

**Authors:** Patricia Ferguson Reyher, Jessica H. Voit

**Affiliations:** ^1^ Department of Internal Medicine, Division of Geriatric Medicine UT Southwestern Medical Center Dallas Texas USA

**Keywords:** delirium, hospital elder life program, REDCap, volunteers

## Abstract

Illustration of the UTSW Workflow AGS Co‐Care HELP WAG App.

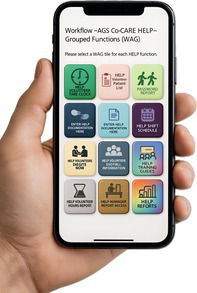

## 1 Introduction

The American Geriatrics Society (AGS) CoCare: Hospital Elder Life Program (HELP) provides an evidence‐based, nonpharmacological model to prevent delirium through targeted interventions delivered by trained volunteers [1, 2, 3]. The University of Texas Southwestern Medical Center (UTSW) utilizes undergraduate students as HELP volunteers [4], however, operational inefficiencies, such as fragmented scheduling, inconsistent documentation, and limited communication pathways, create barriers to optimal workflow. Similar challenges have been identified in other healthcare volunteer programs [5]. To centralize HELP operations, we developed the Workspace App Geriatrics (WAG App) using a secure, REDCap‐based digital platform that meets HIPAA compliance standards. This study describes the implementation of the WAG App and evaluates its impact on volunteer workflow and communication.

## 2 Methods

**FIGURE 1:**
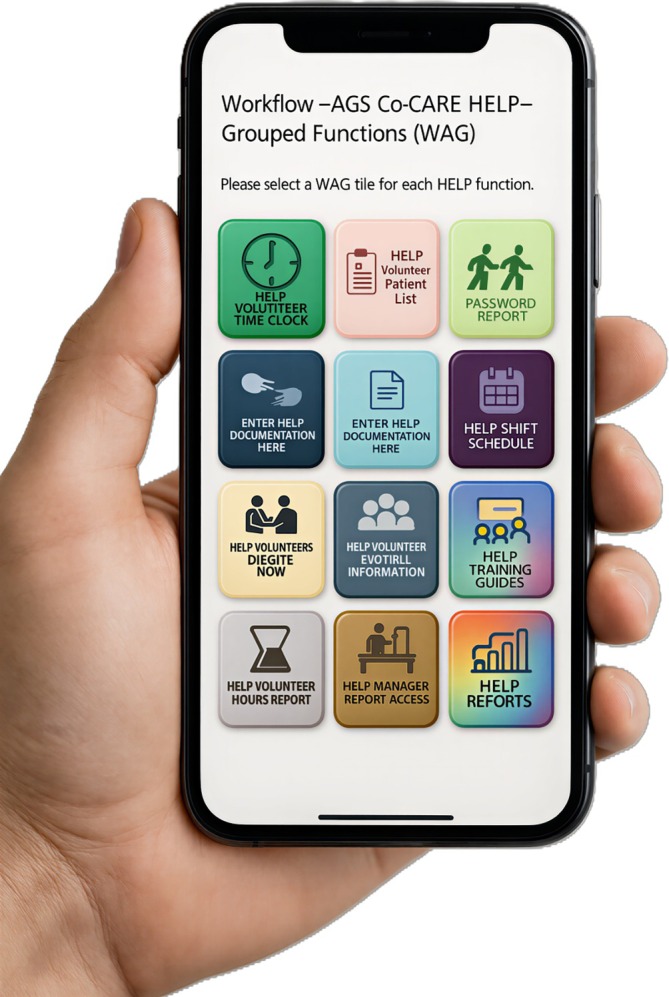
Illustration of the UTSW Workflow AGS Co‐Care HELP WAG App. This demonstrates the WAG App with the 12 grouped tiles that coordinate volunteer care, communication, and educational resources.

The WAG App interface includes 12 workflow tiles supporting real‐time coordination, documentation, scheduling, communication, and administrative oversight (Figure 1). The WAG App centralizes HELP volunteer workflow through real‐time task assignments, access to HELP protocols, integrated training resources, built‐in shift scheduling, and a live view of onsite volunteers. To protect patient‐related information, the WAG App is built with GPS‐based location verification safeguards that only allow access to certain information when volunteers are physically at the hospital. Dashboards that track volunteer activity strengthen workflow coordination. In‐app messaging facilitates real‐time communication between HELP program managers and volunteers.

Volunteers deliver nonpharmacological interventions aligned with AGS CoCare: HELP guidelines, including cognitive orientation, conversation, sleep enhancement strategies, sensory support, hydration cueing, and structured tailored therapeutic activities [1]. Volunteers use the Passdown Report section to record interventions and patient preferences. Students review prior Passdown Reports before visiting patients, providing shift‐to‐shift continuity and highlighting the value of effective handoffs.

Data for this evaluation were drawn from two sources: (a) REDCap Passdown Reports, which captured the number of reports, unique patients, and frequency of entries per patient, and (b) an anonymous REDCap survey distributed via email to active HELP volunteers that assessed workflow efficiency, communication, preparedness, and comparisons to pre‐WAG processes (Supplemental Survey UTSW REDCap S1).

This project was reviewed by the UT Southwestern Human Research Protection Program (HRPP) and determined to be non‐regulated research.

## 3 Results

From July 27, 2023, to March 24, 2024, a total of 927 WAG App patient encounters were documented for 283 patients. Ninety‐five patients had one volunteer encounter documented; 188 patients had multiple HELP volunteer encounters documented over the course of their hospitalization, with an average of four Passdown Reports per patient. The survey achieved a 74% response rate (*n* = 42) (Supplemental Survey UTSW REDCap S1). Among respondents who had volunteered prior to the initiation of WAG (*n* = 24), 100% reported that the WAG App “greatly improved” their delirium‐prevention visits, and 100% responded that the WAG App enhanced their preparedness for patient interactions (Supplemental Table S2). Volunteers consistently highlighted feeling better prepared or more comfortable entering patients' rooms due to the WAG App. For instance, one volunteer shared that the “passdown helps me prepare for meaningful conversations.” Another volunteer commented, “I usually start my shifts by looking at the passdown reports. It helps me get an idea of things to bring up in conversation and which patients would really need visits … I was able to use the passdown report to find out that I and the patient [sic] had a common interest, and I was able to really connect with them on that during our conversation!”

## 4 Discussion

The WAG App transformed the operational structure of UTSW HELP by consolidating scheduling, documentation, communication, and oversight into a secure platform. Prior to the implementation of the app, volunteers were not easily able to communicate with each other. The Passdown Report improved shift‐to‐shift continuity and gave volunteers quick access to patient preferences. This made volunteers better prepared for visits and more comfortable engaging with patients. The WAG App also allows program leadership to easily track volunteer activity, such as the number of patient encounters and volunteer hours. Since the WAG App is built on REDCap, it provides a secure opportunity for further data collection and research.

Limitations of this study include the 74% response rate, as results may reflect bias if students who find less benefit chose not to participate. Although volunteers engaged well with Passdown Reports, the study did not aim to assess behavior changes. Future work is indicated to evaluate whether the WAG App influences volunteer actions.

The WAG App improved HELP program coordination and continuity of care, allowing workflows to adjust rapidly based on patient volume and volunteer availability. Future implications extend beyond HELP, as its adaptable design supports any initiative that requires real‐time alignment of volunteers or trainees to promote efficient, sustainable, and patient‐centered care.

## Author Contributions

Patricia Reyher conceptualized and developed the WAG App, designed the study, led data collection, conducted the analysis, and drafted the manuscript. Jessica Voit provided clinical oversight and program direction and participated in manuscript revision. Both authors reviewed and approved the final manuscript.

## Funding

The authors have no sponsor for this project. Special thanks to the institutions partnering to create the HELP program at UT Southwestern: UT Southwestern Medical Center, Clements University Hospital, and the UT Dallas Hobson Wildenthal Honors College.

## Disclosure

The authors have nothing to report.

## Conflicts of Interest

The authors declare no conflicts of interest.

## Supporting information


**Supplemental Survey UTSW REDCap S1:** HELP WAG App Implementation Survey.
**Supplemental Table S2:** HELP Volunteer Survey Responses.
**Supplemental Survey Response Question 4 Text S3:** HELP Volunteer Survey Comments Section.
