# Supplementary material for: Building and Utilizing a Digital Platform to Strengthen Preparedness of Undergraduates Volunteering With the Hospital Elder Life Program
Source: J Am Geriatr Soc. 2026 Apr 15;74(7):2130–2. doi: 10.1111/jgs.70436 (PMC13418688; doi:10.1111/jgs.70436)
Supplement: Supplementary file 1 — Supplemental Survey UTSW REDCap S1: HELP WAG App Implementation Survey. Supplemental Table S2: HELP Volunteer Survey Responses. Supplemental Survey Response Question 4 Text S3: HELP Volunteer Survey Comments Section. [file JGS-74-2130-s001.pdf]

Supplemental Survey UTSW REDCap, S1: HELP WAG App Implementation Survey

Supplemental Table, S2: HELP Volunteer Survey Responses

Supplemental Survey Response Question 4 Text, S3: HELP Volunteer Survey Comments Section

Supplemental Survey UTSW REDCap, S1:

HELP WAG App Implementation Survey

WAG Pass Down Report Opinion of Value Survey

A A A

The survey information obtained is randomized and anonymous.

Date and Time

[ ] M-D-Y H:M

Were you a participant in the UTD Comets HELP program prior to the implementation of the WAG App?

* must provide value

- ○ Yes
- ○ No

How would you describe the impact of the WAG Pass Down Report on your preparedness for patient visits?

- ○ Greatly improved my patient visit preparedness due to being informed about the patient's state of being and desires.
- ○ Slightly improved my patient visit preparedness due to being informed about the patient's state of being and desires.
- ○ Not improved my patient visit preparedness.

Do you find the WAG App useful for guiding and preparing you for HELP delirium prevention tasks during your shift?

* must provide value

- ○ Yes
- ○ No

Please share an example of a time when information from the WAG Pass Down Report helped you feel more prepared for a patient visit.

* must provide value

Submit

Supplemental Table, S2

HELP Volunteer Survey Responses

**Data Exports, Reports, and Stats**<br>**Number of results returned: 42**<br>**Total number of records queried: 42**<br>**HELP Volunteer Survey (WAG App) Implementation Responses**			
**Were you a participant in the UTD Comets HELP program prior to the implementation of the WAG App?**	**How would you describe the impact of the WAG Passdown Report on your preparedness for patient visits?**	**Do you find the WAG App useful for guiding and preparing you for HELP delirium prevention tasks during your shift?**	**Record ID**
**Yes (1)**	**Greatly improved my patient visit preparedness due to being informed about the patient's state of being and desires. (1)**	**Yes (1)**	**1**
**Yes (1)**	**Greatly improved my patient visit preparedness due to being informed about the patient's state of being and desires. (1)**	**Yes (1)**	**2**
**No (0)**		**Yes (1)**	**3**
**Yes (1)**	**Greatly improved my patient visit preparedness due to being informed about the patient's state of being and desires. (1)**	**Yes (1)**	**4**

**Yes (1)**	**Greatly improved my patient visit preparedness due to being informed about the patient's state of being and desires. (1)**	**Yes (1)**	**5**
**No (0)**		**Yes (1)**	**6**
**Yes (1)**	**Greatly improved my patient visit preparedness due to being informed about the patient's state of being and desires. (1)**	**Yes (1)**	**7**
**No (0)**		**Yes (1)**	**8**
**Yes (1)**	**Greatly improved my patient visit preparedness due to being informed about the patient's state of being and desires. (1)**	**Yes (1)**	**9**
**Yes (1)**	**Greatly improved my patient visit preparedness due to being informed about the patient's state of being and desires. (1)**	**Yes (1)**	**10**
**Yes (1)**	**Greatly improved my patient visit preparedness due to being informed about the patient's state of being and desires. (1)**	**Yes (1)**	**11**
**No (0)**		**Yes (1)**	**12**
**No (0)**		**Yes (1)**	**13**
**Yes (1)**	**Greatly improved my patient visit preparedness due to being informed about the patient's state of being and desires. (1)**	**Yes (1)**	**14**
**Yes (1)**	**Greatly improved my patient visit**	**Yes (1)**	**15**

	preparedness due to being informed about the patient's state of being and desires. (1)		
No (0)		Yes (1)	16
Yes (1)	Greatly improved my patient visit preparedness due to being informed about the patient's state of being and desires. (1)	Yes (1)	17
No (0)		Yes (1)	18
Yes (1)	Greatly improved my patient visit preparedness due to being informed about the patient's state of being and desires. (1)	Yes (1)	19
No (0)		Yes (1)	20
No (0)		Yes (1)	21
Yes (1)	Greatly improved my patient visit preparedness due to being informed about the patient's state of being and desires. (1)	Yes (1)	22
Yes (1)	Greatly improved my patient visit preparedness due to being informed about the patient's state of being and desires. (1)	Yes (1)	23
No (0)		Yes (1)	24
Yes (1)	Greatly improved my patient visit preparedness due to being informed about the patient's state of being and desires. (1)	Yes (1)	25
No (0)		Yes (1)	26

**Yes (1)**	**Greatly improved my patient visit preparedness due to being informed about the patient's state of being and desires. (1)**	**Yes (1)**	**27**
**Yes (1)**	**Greatly improved my patient visit preparedness due to being informed about the patient's state of being and desires. (1)**	**Yes (1)**	**28**
**Yes (1)**	**Greatly improved my patient visit preparedness due to being informed about the patient's state of being and desires. (1)**	**Yes (1)**	**29**
**Yes (1)**	**Greatly improved my patient visit preparedness due to being informed about the patient's state of being and desires. (1)**	**Yes (1)**	**30**
**No (0)**		**Yes (1)**	**31**
**Yes (1)**	**Greatly improved my patient visit preparedness due to being informed about the patient's state of being and desires. (1)**	**Yes (1)**	**32**
**No (0)**		**Yes (1)**	**33**
**Yes (1)**	**Greatly improved my patient visit preparedness due to being informed about the patient's state of being and desires. (1)**	**Yes (1)**	**34**
**No (0)**		**Yes (1)**	**35**
**Yes (1)**	**Greatly improved my patient visit**	**Yes (1)**	**36**

	**preparedness due to being informed about the patient's state of being and desires. (1)**		
**No (0)**		**Yes (1)**	**37**
**No (0)**		**Yes (1)**	**38**
**No (0)**		**Yes (1)**	**39**
**Yes (1)**	**Greatly improved my patient visit preparedness due to being informed about the patient's state of being and desires. (1)**	**Yes (1)**	**40**
**No (0)**		**Yes (1)**	**41**
**Yes (1)**	**Greatly improved my patient visit preparedness due to being informed about the patient's state of being and desires. (1)**	**Yes (1)**	**42**

Supplemental Survey Response Question 4 Text, S3

HELP Volunteer Survey Comments Section

Data Exports, Reports, and Stats

Number of results returned: 42
Total number of records queried: 42

Please share an example HELP WAG App Survey

Please share an example of a time when information from the WAG Pass Down Report helped you feel more prepared for a patient visit.

**Please share an example of a time when information from the WAG Pas ... prepared for a patient visit.** describe_pdrbetter
Knowing before hand through the pass down report about a patient stating that they are in pain, but likes to quietly watch TV and if feeling alone allowed me to begin the visit with understanding and the patient thanked me for spending time with them.
I feel like I know something about the patient before I meet them for the first time. This helps me feel more calm meeting with patient I have never met before.
The pass down reports are especially helpful if the patient speaks another language, is discharged, or is unwilling to speak to volunteers. On other occasions, it prepares me for the specific state of the patient - if they are agitated, friendly, etc.
One pass down report mentioned a patient who was the leader of a group of senior citizens who got together to travel and do mountain biking in various countries around the world. When I visited the patient, I found out he had recently visited Italy, as I had done the summer before. We bonded over the different places in Italy we visited and shared pictures of our travels in Rome and Venice. He showed a picture of a bike ride he organized in Amalfi which looked exceedingly beautiful.
I usually start my shifts by looking at the pass down reports. It helps me get an idea of things to bring up in conversation and which patients would really need visits. One time I was looking at the pass down report and a patient that was mentioned loved travelling and was really lonely. I came in with my partner and the pt seemed agitated but after mentioning travel, the pt opened up and was glowing when we left.
I was able to use the pass down report to find that I and the patient had a common interest, and I was able to really connect with them on that during our conversation!
Gives good insight as to what to expect (if a patient is receptive to volunteers or not, what they enjoy talking about, a heads up about a patient being discharged, etc.) I always read the pass down report especially if volunteers indicate a patient should be seen again, so that I can make sure to prioritize those patients during my shift.
I was able to see that a patient had trouble talking so I made sure that when I talked with the patient to stay close to the patient so I could hear what they had to say
It helped me know what topics to approach in a conversation and what to avoid so that I would not bring up something sensitive to the patient.
I had one patient who was a violinist and was on the quieter side. I also play violin, and this helped me bring her out of her shell and engage with her. The pass down report mentioned this and I was able to use that info in a meaningful way.
WAG informed me of the patient's hobby of art and sports which helped to have a good conversation.
I was able to know where to direct the conversation and keep the patient attentive, since the pass down report indicated their passions/circumstances. It was also made known to me that the patient had been visited once that day already, so it would be optimal for me to be the one to talk more (patient might be slightly exhausted from previous companionships).
The WAG Pass Down Report has offered conversation topics that the patients enjoy plenty of times. I feel better prepared to make them feel cared for with the report.
The pass down reports are very helpful in knowing what to talk to patients about. This can get them excited and make the conversation more engaging. One pass down report mentioned the patient's birthday video, so I ended up asking her about it and watching it with her. They're also helpful in knowing who not to approach/who needs space that day to avoid the awkwardness of that interaction.
Knowing when to visit a patient (ex. some patients don't want visitors for the entire day)
I learned from the pass-down report that a certain patient liked to talk about her pets. This allowed me to connect with her interests and engage her in conversation for longer.
I start my shift by reading the Passdown Report because it helps me know what topics might make the patient feel comfortable. One time, I saw that a patient loved gardening. When I visited, I asked about their favorite flowers, and they lit up and shared stories about their garden. It completely changed the tone of the visit.
The passdown helps me prepare for meaningful conversations. I noticed one patient enjoyed old movies, so I brought up a classic film during the visit. They became so animated talking about their favorite actors that it turned a quiet visit into a lively one.
